# The impact of cardiovascular risk factors on the site and extent of coronary artery disease

**DOI:** 10.5830/CVJA-2011-052

**Published:** 2012-05

**Authors:** AF Zand Parsa, H Ziai, L Haghighi

**Affiliations:** Division of Cardiology, Imam Khomeini Medical Center, Tehran University of Medical Sciences, Tehran, Iran; Division of Cardiology, Imam Khomeini Medical Center, Tehran University of Medical Sciences, Tehran, Iran; Shahid Akbari Hospital, Iran University of Medical Sciences, Tehran, Iran

**Keywords:** coronary artery disease, coronary angiography, proximal versus distal stenosis, cardiovascular risk factors

## Abstract

**Background:**

In patients with coronary artery disease (CAD), the site and extent of coronary artery involvement in terms of proximal versus distal stenosis and multi- versus single-vessel disease have a crucial effect on patients’ outcome. This study was designed to evaluate the relationship between cardiovascular risk factors and the site and extent of coronary artery involvement.

**Methods:**

In this study of patients who had undergone coronary angiography in our hospital, 125 with proximal lesions were enrolled as the case group (group 1) and an equal age-and gender-matched number of patients with non-proximal lesions were selected as the control group (group 2). The two groups were compared based on the presence or absence of diabetes mellitus (DM), hypercholesterolaemia, hypertriglyceridaemia, hypertension (HTN) and cigarette smoking.

**Results:**

The frequency of DM was 33.6 and 10.4% in the case and control groups, respectively, which was statistically significant (*p* < 0.0001). However, the frequency of hypercholesterolaemia in the case and control groups was 30.4 and 29.6% (*p* = 0.89), respectively; for hypertriglyceridaemia it was 19.2 and 16.8% (*p* = 0.062), respectively; for HTN it was 33.6 and 28.8% (*p* = 0.4), respectively; and for cigarette smoking it was 28.8 and 39.2% (*p* = 0.08), respectively, which were not statistically significant. Diabetic patients compared to non-diabetics had more multi-vessel disease (89.1 vs 61%, *p* < 0.0001, respectively), which was statistically significant. There was no relationship between hypercholesterolaemia, hypertriglyceridaemia, HTN and cigarette smoking and extent (multi-vessel involvement) of CAD (*p* = NS).

**Conclusion:**

Proximal and multi-vessel involvement of the coronary arteries in patients with CAD was related to a history of DM but not of hypercholesterolaemia, HTN, cigarette smoking and hypertiglyceridaemia.

## Abstract

Coronary artery disease (CAD) is one of the leading causes of morbidity and mortality worldwide. As atherosclerotic CAD is a heterogeneous disease in terms of severity, extent and site of involvement, these are the most important predictors of outcome of patients with coronary artery disease. The main question is whether or not these heterogeneities have any relationship with cardiovascular risk factors, and if so, which is responsible for which kind of lesion.

Although in some studies a relationship between diabetes mellitus (DM)^1^-^5^ and hyperlipidaemia,^4^-^6^ and severity of CAD has been reported, these studies were focused on the severity of lesions according to the scoring system used and not to the site of lesion in terms of proximal versus distal stenosis. In our study we tried to evaluate the impact of major cardiovascular risk factors such as DM, hypercholesterolaemia, hypertriglyceridaemia, hypertension (HTN) and cigarette smoking on the site and extent of coronary artery involvement in terms of proximal versus distal and multi- versus single-vessel disease.

## Methods

This study was a prospective case–control study that included patients who had undergone coronary angiography in our hospital. Patients with normal coronary arteries were excluded from the study. The case group included 125 patients with significant proximal coronary artery stenosis (≥ 50% luminal narrowing) and the control group included 125 patients with significant non-proximal stenosis, and matched with case group regarding gender and age.

Coronary angiography of all patients was re-evaluated by an expert cardiologist and in the case of controversy, by two experts who were not aware of the patients’ risk factors or other clinical conditions. The two groups were compared for major cardiovascular risk factors such as hyperlipidaemia, HTN, DM and cigarette smoking.

## Statistical analysis

SPSS version 11.5 was used for analysing the data. The Student’s *t*-student test and Chi-square test were used for numerical and continuous variables, respectively. For evaluating data, odds ratio (OR) with 95% confidence interval (CI) were used and *p* > 0.05 was considered significant.

## Results

In the case group 87 patients (69.6%) and in the control group 95 patients (76%) were male. The mean age in the case and control groups was 59.6 ± 10.8 and 58.8 ± 10.9 years, respectively. Regarding gender and age, there was no significant difference between the two groups (*p* = 0.556 and *p* = 0.256, respectively). Clinical and demographic characteristics of patients are presented in Table 1.

**Table 1:** Clinical And Demographic Characteristics Of Patients

*Demographic characteristics*	*Case group n = 125 (100%)*	*Control group n = 125 (100%)*	p-*value*
Males, *n* (%)	87 (69.6)	95 (76)	NS
Age mean ± SD (years)	59.6 ± 10.8	58.8 ± 10.9	NS
Diabetes mellitus, *n* (%)	42 (33.6)	13 (10.4)	< 0.0001
Hypertension, *n* (%)	42 (33.6)	36 (28.8)	NS
Hypercholesterolaemia, *n* (%)	38 (30.4)	37 (29.6)	NS
Hypertriglyceridaemia, *n* (%)	24 (19.2)	21 (16.8)	NS
Cigarette smoking, *n* (%)	36 (28.8)	49 (39.2)	0.08

**Fig. 1.**
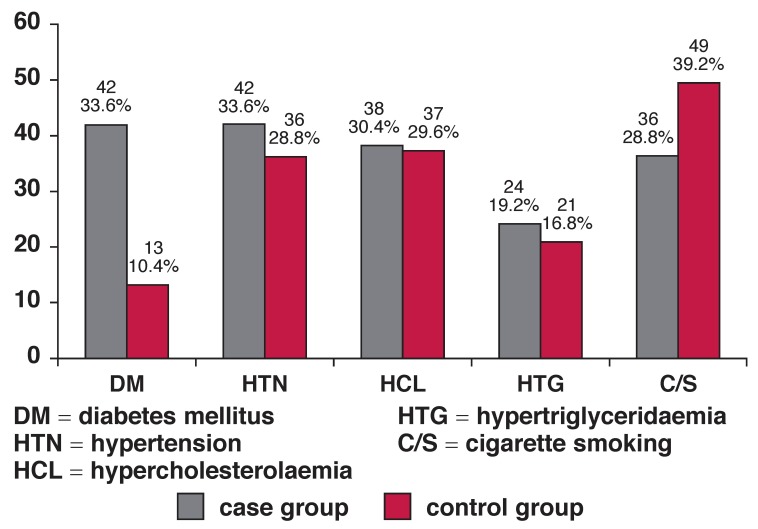
Frequency of cardiac risk factors in the two groups.

Diabetes mellitus was more prevalent in the case group than the control (33.6 vs 10.4%, *p* < 0.0001, OR = 4.36; 95% CI: 2.2–8.6, respectively) and it was statistically significant. There was no difference between the two groups regarding HTN (OR = 1.25; 95% CI: 0.732–2.14, *p* = 0.41), hypercholesterolaemia (OR = 1.04; 95% CI: 0.60–1.78, *p* = 0.86), hypertriglyceridaemia (OR = 1.18; 95% CI: 0.62–2.25, *p* = 0.62) and cigarette smoking (OR = 0.63; 95% CI: 0.37–1.06, *p* = 0.08). The frequency of cardiovascular risk factors in both groups is presented in Fig. 1.

Regarding the extent of CAD, multi-vessel disease was more frequent than single-vessel disease in the diabetics than the non-diabetics (89.1 and 10.9% vs 61 and 39%, respectively, *p* < 0.0001). In hypercholestrolaemic compared to non-hypercholestrolaemic patients, the trend was in favour of more multi-vessel disease but the difference was not statistically significant (76 and 24% vs 63.4 and 36.6%, respectively, *p* = 0.052). Hypertriglyceridaemia, HTN and cigarette smoking had no impact on the extent of coronary artery involvement in terms of multi-vessel versus single-vessel disease. The relationship of these cardiovascular risk factors to the extent of coronary artery involvement is presented in Table 2.

**Table 2:** Relationship Between Cardiovascular Risk Factors And Extent Of Coronary Artery Disease

	*Extent of CAD*		
*Subgroups*	*Single-vessel disease n (%)*	*Multi-vessel disease n (%)*	p-*value*
Diabetic	6 (10.9)	49 (89.1)	< 0.0001
Non-diabetic	76 (39)	119 (61)	
Hypertensive	20 (25.6)	58 (74.4)	NS
Non-hypertensive	62 (36)	110 (64)	
Hypercholesteraemic	18 (24)	57 (76)	0.052
Non-hypercholestraemic	64 (36.6)	111 (63.4)	
Hypertriglyceridaemic	12 (26.7)	33 (73.3)	NS
Non-hypertriglyceridaemic	70 (34.1)	135 (65.9)	
Cigarette smoker	31 (36.5)	54 (63.5)	NS
Non-cigarette smoker	51 (30.9)	114 (69.1)	

## Discussion

Although the relationship between cardiovascular risk factors and CAD has held investigators’ attention for a long time, there are no clear data regarding the impact of risk factors on the site, extent and complexity of coronary artery involvement in terms of proximal or distal and diffuse or segmental involvement. Most studies that have been conducted in this regard were based on index of atheroma burden and extension score of CAD in patients with DM. In the majority of these studies, a strong relationship between DM and increased index of atheroma burden and extension score have been reported.^1^,^2^,^4^,^5^,^7^ However Pajumen *et al*. in their study did not find any relationship between diabetes and extent of CAD compared to non-diabetics.^8^

Uddin *et al*.^1^ and Synkija *et al*.^2^ studied site of coronary artery involvement (proximal versus distal involvement) in diabetic patients versus non-diabetics. Although the trend was towards proximal involvement in diabetic patients, it was not statistically significant (*p* > 0.05).

Synkija reported more multi-vessel disease in hypertensive patients than in non-hypertensives (*p* < 0.0003).^2^ Hong *et al*.^9^ also reported more multi-vessel than single-vessel disease in hypertensive patients (*p* < 0.01). However, Sposito *et al*.^10^ did not find any relationship between hypertension and extent of coronary artery involvement (multi-vessel disease), which was similar to what we found in our study.

Synkija *et al*.^2^ and Sposito *et al*.^10^ found no relationship between hypercholesterolaemia and extent of CAD. Syvanne *et al*.,^5^ Kosuge *et al*.^6^ and Hong *et al*.^9^ reported a relationship between low-density lipoprotein cholesterol and total cholesterol:high-density lipoprotein cholesterol ratio and extent of coronary artery involvement, based on ABI or index of extent of artery involvement (*p* = 0.027, *p* = 0.01 and *p* < 0.01, respectively), but not to the site of coronary artery involvement (proximal versus distal).

In the study of Sposito *et al*.,^10^ post-menopausal women with hypertrygliceridaemia had more extensive CAD compared to those without hypertrygliceridaemia (*p* = 0.0013). Wilson *et al*.^11^ reported more extensive coronary artery involvement in smokers compared to non-smokers based on score of extent of artery involvement (*p* < 0.005), which was opposite to what we found in our study.

None of these studies assessed relationship between hypertriglyceridaemia or cigarette smoking and site of coronary artery stenosis (proximal versus distal stenosis). In our study the trend was towards distal stenosis in smokers compared to non-smokers but it was not statistically significant (*p* = 0.08). Also we did not find a relationship between hypertriglyceridaemia and hypertension and the site of coronary involvement (*p* = NS).

Uddin *et al*.^1^ and Synkija *et al*.^2^ were the only investigators who considered relative frequency of proximal versus distal involvement in their patients. According to their findings, proximal involvement was more frequent in diabetic patients, but it was not statistically significant (*p* > 0.05). In our study, not only frequency of proximal stenosis but also frequency of multi-vessel involvement was significantly higher in diabetics than in non-diabetics (*p* < 0.0001).

## Conclusion

From our findings, proximal involvement of the coronary arteries and more extensive coronary artery disease (multi-vessel disease) were strongly related to a history of DM, but less so to a history of hypercholesterolaemia, and not to a history of hypertriglyceridaemia, hypertension and cigarette smoking.
